# Hyperuricemia, Elevated Body Mass Index, Female Sex, and Albuminuria Increase the Probability of Elevated High-Sensitivity C-Reactive Protein: Results From the National Health and Nutrition Examination Survey 2015–2018

**DOI:** 10.3389/fpubh.2021.689219

**Published:** 2021-08-12

**Authors:** Cristin D. W. Kaspar, Juan Lu

**Affiliations:** ^1^Children's Hospital of Richmond, Virginia Commonwealth University, Richmond, VA, United States; ^2^Division of Epidemiology, Department of Family Medicine and Population Health, Virginia Commonwealth University, Richmond, VA, United States

**Keywords:** hyperuricemia, National Health and Nutrition Examination Survey, inflammation markers, elevated BMI, sex, C-reactive protein

## Abstract

**Importance:** High uric acid (UA) is hypothesized to worsen kidney and cardiovascular disease morbidity *via* activation of systemic inflammation. Clinical trials of UA modification report reduction of the inflammatory marker high sensitivity C-reactive protein (hs-CRP) as an outcome measure, but studies have not demonstrated that hyperuricemia independently increases hs-CRP when adjusted for important confounders such as body mass index (BMI), sex, and age.

**Objective:** To identify clinical risk factors for elevated hs-CRP, including but not limited to hyperuricemia, through a cross-sectional analysis of the National Health and Nutrition Examination Survey (NHANES) 2015–2018.

**Results:** In the final multivariate logistic regression model, the exposure with the strongest effect on the odds of elevated hs-CRP was BMI in the fourth quartile, OR = 13.1 (95% CI 6.25–27.42), followed by female sex (OR = 4.9, 95% CI 2.92–8.34), hyperuricemia (OR = 2.2, 95% CI 1.36–3.45), urine albumin creatinine ratio (ACR; OR = 1.5, 95% CI 1.09–2.18), poor overall health (OR = 1.4, 95% CI 1.18–1.58), and interactions between hyperuricemia and sex (OR = 1.4, 95% CI 1.05–1.83), and between BMI and sex (OR = 1.2, 95% CI 1.03–1.47). Notably, chronic kidney disease (CKD) and CKD surrogates were not associated with hs-CRP despite urine ACR maintaining a significant independent effect.

**Conclusions:** In this national population-based study, we demonstrated that hyperuricemia significantly increases the odds of elevated hs-CRP, independent from BMI, female sex, urine ACR, and overall health status. Further study is recommended to better understand the sex difference in this association and the role of albuminuria, but not CKD, in systemic inflammation.

## Introduction

Recent epidemiologic studies have found that elevated serum uric acid (UA), or hyperuricemia, is independently associated with hypertension, acute kidney injury, and both accelerated decline and increased mortality of patients with chronic kidney disease (CKD) (1–4). One mechanism by which hyperuricemia is proposed to contribute to morbidity and mortality is through direct activation of inflammation. This proinflammatory response occurs below the historically-accepted definition of hyperuricemia, which is a sex-specific serum UA level i.e., associated with clinical gout disease and around the point of supersaturation of UA in serum >7 mg/dl (5–9). Both soluble and crystalline UA activate the Nod-like receptor proteinase 3 (Nlrp3) inflammasome, which triggers the systemic release of proinflammatory cytokines interleukin (IL)-1β, IL-18, and IL-6 (10–16). Indeed, recent studies have shown deleterious effects of UA utilizing a lower definition of hyperuricemia, UA ≥5.5 mg/dl (1, 17–19). The measurement of inflammation *in vitro* using interleukin and cytokine levels, while highly specific, is limited in the clinical setting due to the expense and availability of specific assays (20, 21). The high sensitivity C-reactive protein (hs-CRP), on the other hand, is an acute phase protein and considered a “downstream” inflammatory molecule released by the liver in response to IL-6 when activated by the Nlrp3 inflammasome and other inflammatory states. Hs-CRP is an inexpensive FDA-approved test and is widely applied in clinical settings as a marker of generalized increased inflammation. As such, it has been applied increasingly in research settings as a surrogate marker of inflammation including in UA reduction clinical trials (19, 22–27). However, no study to our knowledge has associated high UA independently with high hs-CRP. This is a necessary step to demonstrate whether hs-CRP is an appropriate outcome measure in UA reduction trials.

The National Health and Nutrition Examination Survey (NHANES) is an annual survey of the United States population that uniquely combines both survey questionnaires with laboratory and physical exam results. Beginning in 2015, the survey added hs-CRP measurements to the panel of laboratory tests. We, therefore, designed this study of a nationally representative pediatric and adult aged cross-sectional sample of the United States population using NHANES 2015–18 data to identify clinical risk factors for elevated hs-CRP, including but not limited to hyperuricemia [as defined by a serum UA ≥5.5 mg/dl (1, 4)].

## Methods

The National Health and Nutrition Examination Survey utilizes a complex random sampling method to survey about 5,000 Americans per year in a nationally representative and weighted sample. The demographic, physical examination, current health, and kidney health questionnaires, and laboratory result datasets from the 2015–16 and 2017–18 public use files were merged for this analysis, maintaining four-year sampling and survey weight to correct for variance estimation. The first year that included hs-CRP was 2015, and 2017–18 is the most recently released dataset. Variables of interest were those with plausible or hypothetical association with elevated inflammation, such as body mass index (BMI), race/ethnicity, biological sex, self-report of overall health status, CKD by estimated glomerular filtration rate (eGFR), personal history of dialysis or personal history of being told the participant had “weak/failing kidneys” in the prior 12 months, urine albumin to creatinine ratio (urine ACR), and UA. To conduct the investigation, several variables were recoded as detailed in the following ways.

Serum UA was categorized into normal (serum UA <5.5 mg/dl) and elevated (≥5.5 mg/dl) based on previously reported definitions of hyperuricemia (4, 18). Categories of UA low (<2.5 mg/dl), normal (2.5–5.5 mg/dl), high (5.6–7.9 mg/dl), and very high (>8 mg/dl) were used in a chi-square test for trend analysis between UA and hs-CRP. The definition of normal or elevated hs-CRP was referred from the specific chemical analyzer used by the NHANES central laboratory.

The continuous age variable was categorized into five subgroups utilizing the United Nations recommended classification for use in health services and nutrition data (28): youth (<14 years), young adulthood (15–24 years), middle adulthood (25–44 years), older adulthood (45–64 years), and elderly/retirement (≥65 years). Elevated BMI is associated with high UA levels (1) and was included in this analysis as a potential confounder. While the 2015–16 NHANES dataset included a BMI percentile variable that allowed for BMI categorization (underweight, normal, overweight, obese) using recommended age- and gender-specific definitions by the Centers for Disease Control, this variable was not included in the 2017–18 dataset. Therefore, in order to utilize all BMI data in this analysis, we transformed the normally distributed pediatric (age <20 years) and adult (age ≥20 years) data into age group-specific quartiles (1st = 0–24, 2nd = 25–49, 3rd = 50–74, 4th = 75–100th percentiles).

Participants with CKD were defined according to the Kidney Disease Improving Global Outcomes classification as eGFR <60 ml/min/1.73 m^2^ using the appropriate pediatric-modified Schwartz (29) or adult CKD-EPI equations (30). The NHANES datasets also include results of an untimed single spot urine Albumin to creatinine ratio (urine ACR) value, with normal defined as urine ACR <100 and ≥100 mg/g considered abnormally elevated in this analysis. NHANES participants were also asked to self-report on their overall health status, and this variable was used as a surrogate for potential inflammation generating chronic medical conditions. Those participants who self-reported to be in good, very good, or excellent overall health were recategorized as “good health” and those with poor or worse self-reports to be in “poor health.”

### Statistical Analysis

Categorical variables were summarized by observed and weighted frequencies and weighted proportions with corresponding 95% confidence intervals (CI). Crude odds ratios (OR) and 95% CI were calculated for variables of interest associated with the outcome of interest, elevated hs-CRP. A chi-square test for trend analysis was performed on increasing UA strata and elevated hs-CRP. A univariate logistic regression was used to determine if each variable of interest was associated with elevated hs-CRP and accepted into an initial multivariate model if *p* < 0.05. The remaining variables were then entered in a multivariate logistic regression model, and backward selection was used with a criterion to exit the model of *p* > 0.1. The likelihood ratio and score and the Wald tests were used to determine the overall goodness of fit of the model, *p* < 0.05, and if there was a gross lack of fit, then the variables would be transformed. Variables were checked for interaction and interaction terms included in the final model if *p* < 0.05. AIC of different models was compared for final model selection. The adjusted OR and 95% CI of the final multivariate logistic regression model are reported. SAS v.9.4 was used for all statistical analyses.

## Results

The merged 2015 through 2018 datasets include data from 19,225 unique observations. The weighted population mean of hs-CRP was 3.43 (SD = 0.1) and followed a parametric distribution. The weighted population serum UA mean of the hyperuricemic group was μ = 6.61 (SD = 0.02) vs. μ = 4.35 (SD = 0.02) of the normal UA group. A linear relationship existed between the continuous variables of serum UA and hs-CRP, but with minimal correlation (*R*^2^ = 0.008, *p* < 0.001). A test for trend between UA and hs-CRP is shown in Table 1 with a significant positive relationship between increasing strata of UA and odds of elevated hs-CRP (*X*^2^ = 995.81440, *p* < 0.0001).

**Table 1:** Odds ratio test for trend, uric acid, and elevated hs-CRP.

**Uric acid (mg/dl)**	**Normal hs-CRP**	**Elevated hs-CRP**	**Odds ratio**
<2.5	6,977	162	1.00 (reference)
2.5–5.5	5,713	973	7.335
5.6–7.9	3,951	878	9.571
>8	399	172	18.566

The weighted population eGFR mean of the CKD group was μ = 48.80 (SD = 0.64) vs. μ = 87.09 (SD = 0.52) of the non-CKD group. Table 2 includes the summary statistics of several demographic and clinical characteristics of the study population. The observed, weighted frequencies, and weighted proportions with 95% CI are reported for each variable of interest.

**Table 2:** Summary statistics.

	**Observed frequency**	**Weighted frequency**	**Weighted proportion**	**95% CI**	
				**LL**	**UL**
**Age, years**
Youth, 0–14	4,858	54,042,808	0.17	0.17	0.17
Young adulthood, 15–24	2,246	41,439,348	0.13	0.13	0.13
Middle adulthood, 25–44	3,497	83,555,752	0.26	0.26	0.26
Older adulthood, 45–64	3,745	83,773,100	0.26	0.26	0.26
Elder, ≥65	*2,694*	48,249,472	0.15	0.15	0.15
**Biological sex**
Male	8,949	155,701,858	0.49	0.49	0.49
Female	9,299	162,960,025	0.51	0.51	0.51
**Race and ethnicity**
*Hispanic*					
Mexican-American	3,135	34,093,151	0.11	0.11	0.11
Other Hispanic	2,005	22,837,131	0.07	0.07	0.07
*Non-Hispanic, Other*					
White	5,879	190,792,815	0.60	0.60	0.60
Black	4,062	37,887,567	0.12	0.12	0.12
Other/multi-racial	3,167	33,051,219	0.10	0.10	0.10
**Body mass index (percentile)**
Quartile 1 (<25th)	4,185	76,029,474	0.25	0.25	0.25
Quartile 2 (25–50th)	4,299	76,431,703	0.25	0.25	0.25
Quartile 3 (50–75th)	4,095	76,744,288	0.25	0.25	0.25
Quartile 4 (>75th)	4,182	77,823,600	0.25	0.25	0.25
**Chronic kidney disease**
Normal, eGFR ≥ 60	1,885	52,912,190	0.17	0.17	0.17
Abnormal, eGFR <60	16,363	265,749,693	0.83	0.83	0.83
**Personal history of weak/failing kidneys in last 12 months, response**
Yes	438	7,575,786	0.03	0.03	0.03
No	10,286	228,835,674	0.97	0.97	0.97
**Personal history of dialysis in last 12 months, response**
Yes	36	455,102	0.06	0.06	0.06
No	401	7,114,854	0.94	0.94	0.94
**Self-report of overall health**
Poor	2,766	43,939,675	0.14	0.14	0.14
Good	15,482	274,722,207	0.86	0.86	0.86
**Urine albumin to creatinine ratio, mg/g**
Normal <100	17,492	307,057,243	0.96	0.96	0.96
Elevated ≥ 100	756	11,604,639	0.04	0.04	0.04
**Serum uric acid, mg/dl**
Normal <5.5	12,848	207,325,224	0.65	0.65	0.65
Elevated ≥ 5.5	5,400	111,336,659	0.35	0.35	0.35
**High sensitivity C-reactive Protein, mg/L**
Normal <7.48	16,063	276,052,758	0.87	0.87	0.87
Elevated ≥ 7.48	2,185	42,609,124	0.13	0.13	0.13

In Table 3, the crude OR and 95% CI are reported for each variable of interest by the outcome of elevated hs-CRP. The variables “personal history of dialysis in the prior 12 months,” of respondents 20 years and older, and race/ethnicity were not significantly associated with elevated hs-CRP in the crude analysis. CKD, broadly categorized by eGFR <60, age, and personal history of weak/failing kidneys, did not meet the criteria to remain in the multivariate model. Age and race/ethnicity were kept in the multivariate model *a priori* per standard practice. Hyperuricemia, elevated BMI (second to fourth quartiles), female sex, abnormal albuminuria, and overall self-report of poor health significantly increased the odds of having elevated hs-CRP in the univariate analysis.

**Table 3:** Crude and adjusted odds ratio, outcome elevated hs-CRP.

**Variables of Interest**	**Crude**	**95% CI**		***p*-value**	**Adjusted**	**95% CI**		***p*-value**
	**OR**	**LL**	**UL**		**OR**	**LL**	**UL**	
**Age, years**								
Youth, 0–14	1.00				1.00			
Young adulthood, 15–24	3.68	2.66	5.08	<0.001	2.468	1.777	3.429	<0.0001
Middle adulthood, 25–44	6.57	5.34	8.07	<0.001	5.23	4.228	6.468	<0.0001
Older adulthood, 45–64	6.69	5.28	8.48	<0.001	5.003	3.916	6.394	<0.0001
Elderly/retirement, ≥65	4.79	3.77	6.08	<0.001	3.295	2.527	3.295	<0.0001
**Sex**								
Male	1.00				1.00			
Female	1.77	1.52	2.05	<0.001	4.933	2.917	8.341	<0.0001
**Race and ethnicity**								
*Hispanic*					–			
Mexican-American	1.07	0.90	1.27	0.428	1.143	0.851	1.536	0.3621
Other Hispanic	0.99	0.82	1.18	0.887	1.137	0.81	1.596	0.4446
*Non-Hispanic, Other*					–			
White	1.00				1.087	0.807	1.463	0.5734
Black	1.26	1.05	1.51	0.014	1.155	0.865	1.541	0.3167
Other/multi-racial	0.81	0.61	1.07	0.135	1.00			
**BMI category**								
Quartile 1	1.00				1.00			
Quartile 2	1.40	1.11	1.77	0.007	1.866	1.4	2.488	0.0001
Quartile 3	2.16	1.71	2.73	<0.001	3.578	2.111	6.064	<0.0001
Quartile 4	6.63	5.37	8.19	<0.001	13.095	6.254	27.421	<0.0001
**Chronic kidney disease**								
Normal, eGFR ≥60 ml/min/1.73 m^2^	1.00				–			
Abnormal, eGFR <60 ml/min/1.73 m^2^	1.37	1.14	1.65	0.002	–			
**Personal history of weak/failing kidneys in last 12 months**					–			
No	1.00				–			
Yes	1.75	1.22	2.52	0.004	–			
**Personal history of dialysis in last 12 months**					–			
No	1.00				–			
Yes	1.47	0.56	3.88	0.422	–			
**Self-report of health**					–			
Good	1.00				1.00			
Poor	2.35	2.09	2.65	<0.001	1.361	1.177	1.575	0.0002
**Urine albumin:creatinine ratio, mg/g**								
Normal <100	1.00				1.00			
Elevated ≥ 100	1.82	1.42	2.35	<0.001	1.537	1.086	2.175	0.0170
**Serum uric acid, mg/dl**								
Normal <5.5 mg/dl	1.00				1.00			
Elevated ≥ 5.5 mg/dl	1.81	1.58	2.08	<0.001	2.167	1.361	3.448	0.0019
**Interaction serum uric acid and sex**					1.385	1.052	1.825	0.0221
**Interaction BMI and sex**					1.234	1.033	1.473	0.0218

Interactions between UA and urine ACR, sex, BMI, self-report of overall health, and age were checked. Only the interaction between UA and sex, and BMI and sex were statistically significant (*p* < 0.001), and therefore, both were included in the final multivariate model. The final adjusted ORs and 95% CIs are reported in Table 3. All quartiles of BMI above the referenced first quartile significantly increased the odds of elevated hs-CRP, the fourth quartile having the largest effect at 13.1 (CI 6.25–27.42). Female sex increased the odds of elevated hs-CRP by 4.9 (CI 2.92–8.34), and hyperuricemia by 2.2 (CI 1.36–3.45). Interestingly self-report of poor overall health increased odds of hs-CRP by 1.4 (CI 1.18–1.58), and abnormal urine ACR by 1.5 (CI 1.09–2.18).

## Discussion

To our knowledge, this is the first analysis of the association between hs-CRP and hyperuricemia that utilizes a large nationally representative sample of pediatric and adult participants. After adjustment for multiple variables, we found that race/ethnicity was not associated with the outcome. The most significant effect on the odds of elevated hs-CRP was from having a fourth quartile BMI where the odds are 13 times as large as the first quartile BMI. BMI quartiles also held a dose-dependent effect, with the odds of elevated hs-CRP increasing for each level of BMI above the first quartile. This finding is consistent with other studies where obesity is related to markers of increased systemic inflammation (31, 32). Female sex over male sex had the next greatest effect on the odds of elevated hs-CRP, with nearly five times greater odds. A gender difference has been noted by several studies related to poorer outcomes of disease morbidity and mortality, and higher expression of inflammation among women (33–35). The effect of female sex-UA interaction was also demonstrated in one study of adult chronic heart failure patients (36). Hyperuricemia was found to independently increase 5-year all-cause mortality in adults with chronic heart failure only in women and not met. Age held a reverse “U” shaped curve with increased odds overall above pediatric age groups for elevated hs-CRP in the middle age subgroups and lessened odds in the elderly.

Interestingly, CKD, represented in this analysis in multiple ways by eGFR <60, a personal history of weak/failing kidneys, or personal history of dialysis in the last 12 months, was not found to be significant in the adjusted analysis. In the NHANES dataset, the number of positive self-reports for weak/failing kidneys or personal history of dialysis was low, which may have been a limiting factor in this analysis. In contrast, abnormal albuminuria, a condition that develops in some types of CKD, remained significant in the multivariate model, and increased the probability of elevated hs-CRP by 54%. One study found both abnormal urine ACR and CKD (defined similarly to the study analysis) to be associated with biomarkers of inflammation but utilized a smaller and adult-only population (*N* = 3,294) from the Framingham Offspring Cohort (37). Proteinuria is a known accelerator of CKD progression *via* induction of renal tubular inflammatory cell infiltration and fibrogenesis (38, 39). This analysis seems to indicate that proteinuria increases systemic inflammation, but that CKD does not. This difference and the mechanism by which this occurs should be investigated in future studies.

Finally, hyperuricemia, as defined by a serum UA ≥5.5 mg/dl, increased the odds of elevated hs-CRP by 2.17. We expected to find an interaction between BMI and UA; however, the *p*-value for this interaction term was not statistically significant (*p* < 0.05) despite multiple studies suggesting such a relationship (40–42). A possible explanation is that while a diet that leads to UA overproduction such as with high consumption of purines and fructose-containing foods may result in hyperuricemia and an elevated BMI, one may also develop hyperuricemia from renal underexcretion of UA. Renal underexcretion of UA may be secondary to renal dysfunction or genetic polymorphisms in UA transporter proteins (6), causing hyperuricemia independent of BMI. Our uniquely large study population allowed identifying these independent effects, compared with other studies that had smaller and less diverse patient populations, subject to more bias or confounding.

## Conclusions

Using national population-based data, this study demonstrated that hyperuricemia is significantly associated with increased odds of elevated hs-CRP in a dose-dependent manner, but that the greatest effect came from the fourth quartile BMI, female sex, and age, which was consistent with previous studies. Further studies are recommended to better understand the sex difference in this association, the role of albuminuria, but not CKD, in elevated hs-CRP, as well as to further validate the utility of hs-CRP as an outcome measure in UA modification trials.

## Data Availability Statement

The raw data supporting the conclusions of this article will be made available by the authors, without undue reservation.

## Author Contributions

Material preparation, data processing, and analysis were carried out by CK. The first draft of the manuscript was written by CK. All authors commented on the previous versions of the manuscript, contributed to the conception and design of the study, and read and approved the final manuscript.

## Conflict of Interest

The authors declare that the research was conducted in the absence of any commercial or financial relationships that could be construed as a potential conflict of interest.

## Publisher's Note

All claims expressed in this article are solely those of the authors and do not necessarily represent those of their affiliated organizations, or those of the publisher, the editors and the reviewers. Any product that may be evaluated in this article, or claim that may be made by its manufacturer, is not guaranteed or endorsed by the publisher.
